# Esthesioneuroblastoma: one of the causes of proptosis

**DOI:** 10.1186/1746-160X-9-19

**Published:** 2013-07-27

**Authors:** Sajid Ansari, Kaleem Ahmad, Kanchan Dhungel, Mukesh Kumar Gupta, Md Farid Amanullah

**Affiliations:** 1 Department of Radiodiagnosis and imaging, B.P. Koirala Institute of Health Sciences, Dharan, Sunsari 56700, Nepal; 2 Department of Orthopaedics, B.P. Koirala Institute of Health Sciences, Dharan, Nepal

**Keywords:** Esthesioneuroblastoma, Olfactory neuroblastoma, Nasal cavity, Proptosis, Computed tomography

## Abstract

Esthesioneuroblatoma (Olfactory neuroblastoma) is a rare malignant neoplasm arising from the olfactory epithelium with bimodal age distribution between with first peak in second decades and second peak in sixth decade. Proptosis due to esthesioneuroblastoma is one of the rare causes. They have a long natural history characterized by frequent local or regional recurrence. Computed tomography and magnetic resonance imaging are the imaging modalities for diagnosing these tumors. A multidisciplinary approach with surgery and radiation therapy is an excellent treatment options for these tumors with chemotherapy being used to treat advanced or recurrent disease.

## Introduction

Esthesioneuroblastoma (olfactory neuroblastoma) is a rare neuroectodermal malignant neoplasm that originates from the olfactory sensory epithelium [1-3]. It accounts for upto 5.0% of malignant tumors of the nasal cavity. Berger et al. in 1924 was first to describe this disease. The incidence curve for this disease has a bimodal shape with the first peak in the 2nd decade and 2nd peak in 6th decade with roughly equal sex distribution [2]. Patients present with nonspecific symptoms of nasal obstruction, epistaxis, headache, pain, visual disturbances, anosmia. Owing to the nonspecific nature of the presenting symptoms, patients often have a long history prior to diagnosis [3]. Tumors involving the orbital area generally present with epiphora, decreased visual acuity and proptosis [4]. We report a case of esthesioneuroblastoma in 60 years old male who presented with proptosis and nasal obstruction, diagnosed on computed tomography.

## Case report

A 60 years old male presented to with complaints of nasal obstruction, nasal bleeding and bulging of left eye with decreased vision for 1.5 years. He also noticed a rapidly increasing swelling over nasal bridge for 4 months and associated headache and neck pain. There was insignificant past history and family history. On systemic examination, there was loss of smell sensation. Local examination revealed a diffuse, firm, nontender swelling with ill-defined margins over the nasal bridge, glabella and over left maxillary region. There was no rise in local temperature. Pinkish mass was visible in both nostrils with purulent nasal discharge. There was proptosis of left eye and mild lateral deviation with limited eyeball movement; however visual acuity was normal. There is no evidence of lymphadenopathy. Blood profile demonstrates normal limits of complete blood count, renal function test and liver function test.

**Figure 1:**
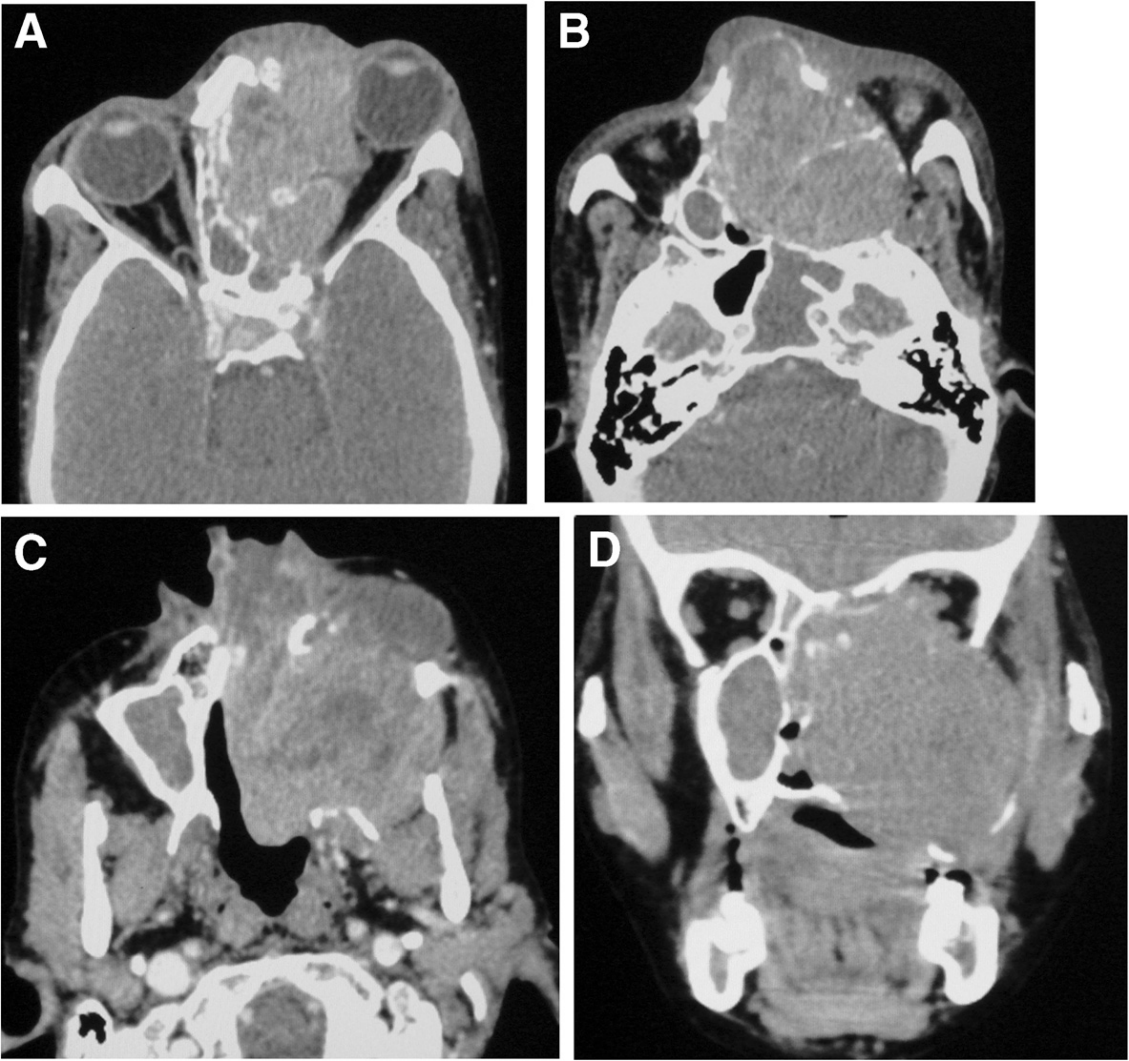
**CT images in soft tissue window showing mass in ethmoid sinus and nasal cavity extending into the left orbit. A** &**B**: Axial section CT image showing heterogeneously enhancing soft tissue density mass in bilateral ethmoid sinuses and both nasal cavities with bony destruction and extension into left orbit causing proptosis. **C** &**D**: Axial and coronal section CT images showing lateral extension of the mass into left infratemporal region with destruction of the postero-lateral wall of left maxillary sinus.

**Figure 2:**
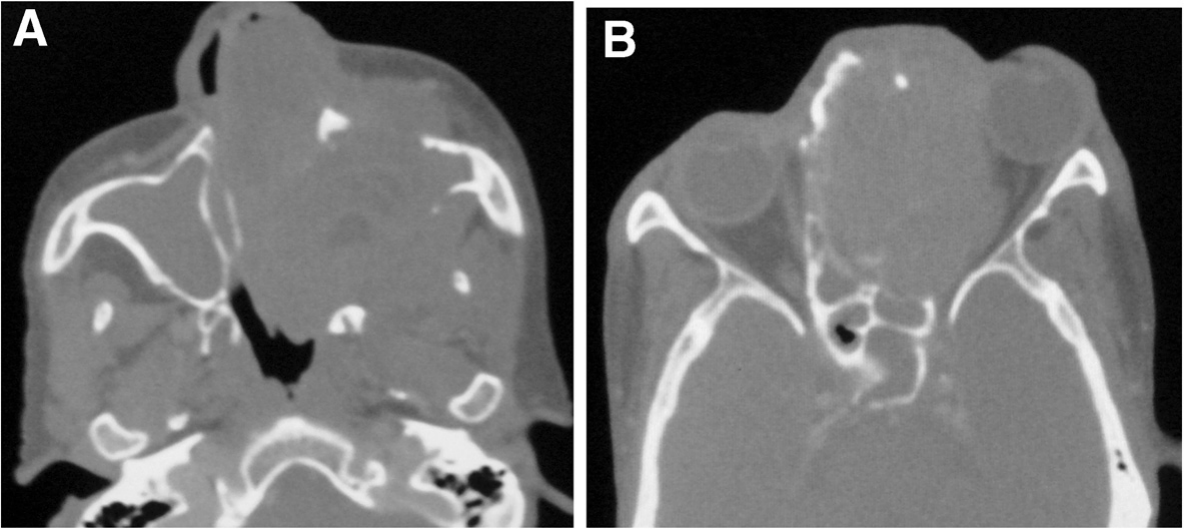
**CT images in bone window showing bony destruction. A** &**B**: Axial section CT images (bone window) showing destruction of medial and postero-lateral wall of left maxillary sinus, left lateral wall of nasal cavity, nasal septum, ethmoid sinus and medial wall of left orbit by the mass.

Computed tomography (CT) scan of paranasal sinuses including orbit was performed in axial section with coronal and sagittal reconstructions. CT revealed large, ill-defined, heterogeneously enhancing soft tissue density mass in both nasal cavities and ethmoid sinuses with destroyed nasal septum (Figure 1A, 1B). There was destruction of medial wall of left maxillary sinus and left lateral nasal wall with extension into left maxillary antrum. Laterally, the mass extended into the left infratemporal region with destruction of the postero-lateral wall of left maxillary sinus (Figure 1C, 1D, 2A). There was also destruction of medial wall of left orbit with intraorbital extension causing antero-latero-inferior displacement of the eyeball resulting in proptosis (Figure 1A, 2B). The mass extended superiorly involving frontal sinuses; however cribriform plate was normal. There was no intracranial extension. Inferiorly, there was destruction of hard palate with intraoral extension (Figure 1C, 1D). Posteriorly, it was extending into nasopharynx through choana and destruction of anterior sphenoid sinus wall extending into sinus (Figure 2A, 2B).

Histopathology revealed monomorphic malignant round cell tumour with rossette formation, consistent with esthesioneuroblastoma. Patient was advised for chemotherapy/radiotherapy and was referred to oncology institute.

## Discussion

Esthesioneuroblastoma has been reported to originate from sphenopalatine ganglion, vomeronasal organ of Jacobson, neuroepithelial cells of the olfactory membrane, the ectopic olfactory epithelium in the nasal mucosa and the amine precursor uptake and decarboxylation cells [1,2]. It is a locally infiltrating malignant neoplasm; frequently invades the skull base, orbit and adjacent soft tissue from the typical “cribriform” site. In our patient, the neoplasm has infiltrated the left orbit through destruction of medial orbital wall resulting in proptosis. Distant and regional metastases are seen in about 14–38% of cases at the time of diagnosis; cervical lymph nodes, lung and bone being the frequently involved sites [4]. Esthesioneuroblastoma should be differentiated from other malignant tumors in anterior skull base.

CT and magnetic resonance imaging (MRI) are helpful to identify the margin and spread approaches of the tumor**.** Fine-cut CT scan (3 mm slice thickness) with direct coronal imaging is the initial radiological study of choice [5]. On CT scan, the tumor usually present with a heterogeneous mass, sometimes with focal necrosis or calcification. The paranasal sinuses and anterior skull base are often destroyed, while the clivus is intact. It appears as isointense on T1-weighted and hyperintense on T2-weighted MR images with heterogeneous enhancement on gadolinium contrast study. MRI help to differentiate tumour from obstructed secretions in paranasal sinuses, determining meningeal and extradural spread and to detect perineural spread [5,6]. Angiography demonstrates tumour blush in majority of cases [6].

There are several staging systems described for olfactory neuroblastoma; Kadish system [7] has been the frequently used staging system with good prognostic correlation. It has divided the tumors in three stages: Stage A- tumors restricted to the nasal cavity; Stage B- tumors involving the nasal cavity and paranasal sinuses and Stage C- tumors extending to beyond the paranasal sinuses (orbit, skull base or metastasis).

Differential diagnosis of esthesioneuroblastoma includes inverting papilloma, squamous cell carcinoma, adenocarcinoma, sinonasal undifferentiated carcinoma, hemangioma and metastasis. In addition to imaging, histopathological correlation is must in making diagnosis [8]. Esthesioneuroblastomas are histologically similar to adrenal or sympathetic ganglionic neuroblastomas and retinoblastomas [9]. On histopathology, the presence of fibrillary intercellular background along with Homer-Wright rosettes is considered to be diagnostic of esthesioneuroblastoma [10].

Open craniofacial resection or endonasal endoscopic resection is done for stage A and B tumors. Chemotherapy is usually implemented in patients with locally advanced, metastatic or recurrent disease [11-14]. The commonly used chemotherapy combinations are cyclophosphamide plus vincristine and cisplatin-based regimens [15]. The combination of surgery and radiotherapy is the most frequent treatment approach with highest cure rates, however definitive radiotherapy as a conservative management is also used. Despite definitive local therapy, local recurrence and distant metastases have been reported with the metastasis being 25.0-50.0% of cases. In patients with recurrent or metastatic olfactory neuroblastoma, chemotherapy is therefore often implemented. Radiotherapy is usually adopted as an adjuvant following surgery, especially for cases with neck metastasis or as a neoadjuvant before surgery [14,16]. Compared with other sinonasal malignancies, the prognosis of esthesioneuroblastoma is much better, with a disease-free survival at 5 years of more than 80.0% [17].

## Conclusion

In conclusion, clinicians should be aware of this malignant disease and proptosis should be considered as one of the differential diagnosis of esthesioneuroblastoma. Computed tomography and magnetic resonance imaging are the modalities of choice in diagnosing, looking for extent and staging of tumor in esthesioneuroblastoma. A multidisciplinary approach with surgery and radiation therapy is the treatment options for these tumors with chemotherapy being used to treat advanced or recurrent disease.

## Consent

Written informed consent was obtained from the patient for publication of this case report and any accompanying images. A copy of the written consent is available for review by the Editor of this journal.

## Abbreviations

CT: Computed tomography; MRI: Magnetic resonance imaging.

## Competing interest

Authors declare no conflict of interest.

## Authors’ contributions

SA has done substantial contributions to conception and design, acquisition of data, Analysis and interpretation of data, drafting the article, revising it critically for important intellectual content and final approval of the version to be published. KA has done analysis and interpretation of data, revising it critically for important intellectual content, final approval of the version to be published. KD has done substantial contributions to conception and design, revising it critically for important intellectual content and final approval of the version to be published. MKG has done substantial contributions to conception and design, revising it critically for important intellectual content and final approval of the version to be published. MFA has done drafting the article, revising it critically for important intellectual content and final approval of the version to be published. All authors read and approved the final manuscript.
